# Metoprolol Dose Equivalence in Adult Men and Women Based on Gender Differences: Pharmacokinetic Modeling and Simulations

**DOI:** 10.3390/medsci4040018

**Published:** 2016-11-15

**Authors:** Andy R. Eugene

**Affiliations:** Division of Clinical Pharmacology, Department of Molecular Pharmacology and Experimental Therapeutics, Gonda 19, Mayo Clinic, 200 First Street SW, Rochester, MN 55905, USA; eugene.andy@mayo.edu; Tel.: +1-507-284-2790

**Keywords:** metoprolol, pharmacokinetics, gender differences, modeling, monolix

## Abstract

Recent meta-analyses and publications over the past 15 years have provided evidence showing there are considerable gender differences in the pharmacokinetics of metoprolol. Throughout this time, there have not been any research articles proposing a gender stratified dose-adjustment resulting in an equivalent total drug exposure. Metoprolol pharmacokinetic data was obtained from a previous publication. Data was modeled using nonlinear mixed effect modeling using the MONOLIX software package to quantify metoprolol concentration–time data. Gender-stratified dosing simulations were conducted to identify equivalent total drug exposure based on a 100 mg dose in adults. Based on the pharmacokinetic modeling and simulations, a 50 mg dose in adult women provides an approximately similar metoprolol drug exposure to a 100 mg dose in adult men.

## 1. Introduction

A recent population pharmacokinetic (popPK) paper used modeling and simulation to recommend dose adjustments for metoprolol, a cardio-selective β-blocker, for geriatric men and women who generally suffer from multiple comorbidities [1]. However, gender-stratified doses in the non-geriatric population not being treated for various ailments have not been established. Therefore, the primary aim of this paper is to identify the doses resulting in an approximately equivalent area under the concentration–time curve (AUC) for men and women administered an oral dose of metoprolol. To do so, this paper will use pharmacokinetic (PK) modeling and simulation to identify the full PK equation parameters and describe metoprolol using a one-compartment model based on data from the well-cited gender-differences study by Luzier and colleagues [2]. 

The original study showed that the total systemic drug exposure of the active enantiomer, *S*-metoprolol, resulted in an AUC of 417 mcg·h/L for men and an AUC of 867 mcg·h/L for women [2]. These results suggest that, on average, women are exposed to more than double the systemic drug metoprolol when compared to men receiving the same 100 mg oral dose. Since this original publication in 1999, no recommended dose adjustments have been made for women despite these gender differences in metoprolol pharmacokinetics. This may have been due to the similarities in the pharmacodynamics represented as the maximum percent decrease in systolic blood pressure and the heart rate reported in the original article. However, a series of publications indicating gender-specific adverse effects such as prolonged hypotension and bradycardia due to gender difference in the cytochrome (CYP) P450 enzymes, when compared to men, occur when using metoprolol [3,4,5,6].

Hence, with this as a foundation, the objective of this article is to identify doses achieving equivalent systemic exposures in men and women administered metoprolol. A secondary aim is to use the PK model parameters to conduct a Clinical Trial Simulation (CTS) of a 100 mg dose of metoprolol. I hypothesize that, considering the differences in physiology between men and women, a 30%–40% dose reduction would be required to normalize systemic exposure to metoprolol.

## 2. Materials and Methods

### 2.1. Dataset

The original concentration–time data used in this dose-finding study are based on the average *R*-metoprolol and *S*-metoprolol data points originating from the Luzier and colleagues’ article published in 1999 [2]. The data were digitized for gender-stratified *R*-metoprolol and *S*-metoprolol resulting in four distinct pharmacokinetic curves. The original study was conducted in 20 healthy study participants (10 men and 10 women) with an age ranging from 20 to 36 years old who received nine total doses of 100 mg of metoprolol every 12 h. A full description may be obtained from the original Luzier and colleagues’ article [2].

### 2.2. Pharmacokinetic Modeling

As a result of the final published manuscript not including the absorption rate constant (Ka) or the absorption lag time (Tlag), a pharmacokinetic fitting of the digitized dataset was performed using the MONOLIX (MOdèles NOn LInéaires à effets miXtes) software (version 4.3.3, Lixoft, Orsay, France). The MONOLIX software uses the Stochastic Approximation Expectation–Maximization (SAEM) algorithm with a Markov Chain Monte-Carlo (MCMC) procedure to compute the maximum likelihood estimates for the final population pharmacokinetic parameters [7,8]. Model validation was based on the goodness-of-fit plots and the precision of digitized data points aligning, using pharmacokinetic simulations of the original study. 

### 2.3. Dose Finding Simulations

Individual pharmacokinetic simulations were conducted using A4S PK/PD Simulator (version 2012) developed by Accelera (Nerviano, Italy) and Pfizer (Sandwich, Kent, UK) and compiled using MATLAB (version 6.5.1.199709, Release 13, Mathworks, Natick, MA, USA) [9]. The A4S PK/PD simulator uses the MATLAB ordinary differential equation (ODE) solver and is used by Pfizer and Accelera project scientists implementing design preclinical PK/PD studies and clinical trial simulations [9].

### 2.4. Clinical Trial Simulations

A clinical trial simulation of 50 men and 50 women administered a 100 mg dose of *S*-metoprolol will be conducted using the R programming language (version 3.2.2, The R Foundation for Statistical Computing, Vienna, Austria) [10]. Metoprolol plasma levels will be simulated for the follow times: 0, 0.1, 0.2, 0.3, 0.4, 0.6, 0.8, 1, 2, 4, 6, 8, 12, 14, 16, 18, and 24 h. To describe the population variability, the coefficient of variations for the total body clearance rate (CL) and the volume (V) of distribution in the central compartment were referenced from the main compartment in the original study. For the total body clearance, the following values were used for the coefficient of variations (CV%): *S*-enantiomer: *S*-CL_men_ = 59%, *S*-CL_women_ = 49% and the *R*-enantiomer: *R*-CL_men_ = 70%, *R*-CL_women_ = 59% [2]. Further, for the oral volume of distribution in the central compartment, the follow values were used for the CV%: *S*-enantiomer: *S*-V_men_ = 44%, *S*-V_women_ = 34% and the *R*-enantiomer: *R*-V_men_ = 52%, *R*-V_women_ = 36% [2]. Since the original study did not publish the absorption rate constant, this analysis will assume a 40% variation.

## 3. Results

### 3.1. Metoprolol Pharmacokinetics

**Figure 1:**
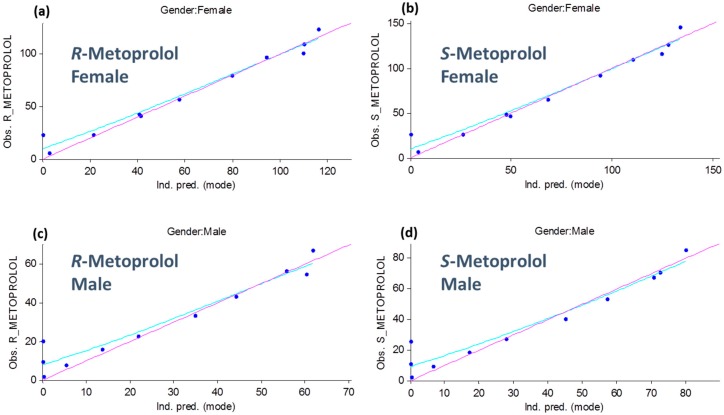
Goodness-of-fit plots for the *R*-metoprolol (**a**) and (**b**) and *S*-metoprolol (**c**) and (**d**) enantiomers for males (**b**) and (**d**) and females (**a**) and (**c**). The *x*-axes depict the predicted plasma levels and the *y*-axes depict the observed metoprolol plasma levels.

Consistent with current publications, a one-compartment model adequately described metoprolol pharmacokinetics [11,12,13]. Estimation of model parameters included absorption rate constant, clearance rate, volume of distribution, and the absorption time lag. The quantified pharmacological properties of *R*- and *S*-metoprolol are summarized in Table 1. Goodness-of-fit plots for the observed versus predicted plasma concentrations are shown in Figure 1. The maximum metoprolol plasma concentrations (Cmax) and the total systemic metoprolol drug exposures, measured as the AUC for males and females are consistent with the original study.

**Figure 2:**
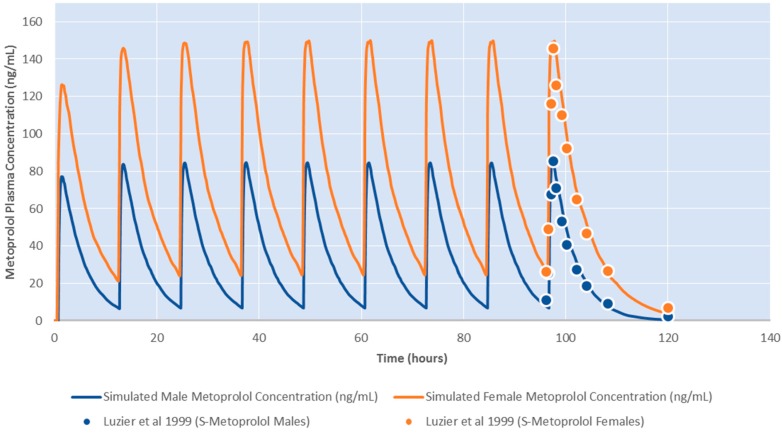
Model parameter validation using pharmacokinetic dosing simulations using the gender-stratified model parameters and the original Luzier et al. experimental plasma concentrations. Female (**orange**—higher line) and male (**blue**—lower line) dosing simulations for the nine 100 mg metoprolol doses illustrate an adequate fit to the experimental results.

Qualification of the final pharmacokinetic model parameter estimates are based on conducting pharmacokinetic simulations according to the original published study protocol [2]. In the original protocol, study participants were administered nine 100 mg doses of metoprolol, so concentration–time simulations of the dosing regimen were performed using the new model results while the original published data points were overlaid to evaluate model precision. The fit results of the simulated metoprolol pharmacokinetics and the original experimental data illustrating an adequate model fit to the original data are shown in Figure 2.

### 3.2. Dose-Finding Simulations

**Figure 3:**
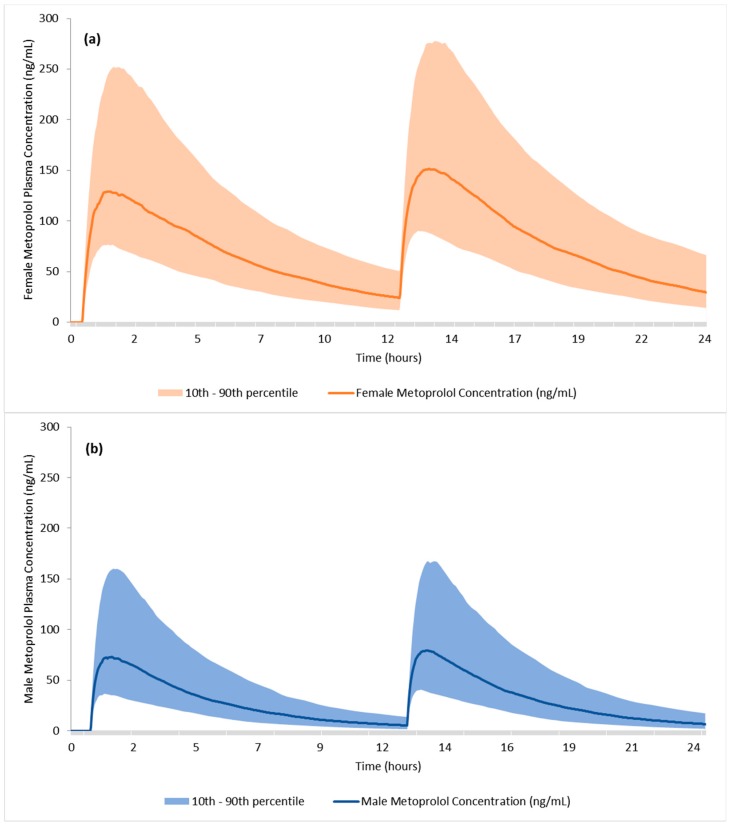
Dose-finding simulation results of two 100 mg doses of metoprolol every 12 h, for (**a**) men and (**b**) women. The results are based on the *S*-metoprolol modeling parameters where the solid lines illustrate the typical value of plasma concentrations and the shaded bands represent the 10th and 90th percent confidence interval for 3000 virtual patients.

Using the one-compartment model parameter estimates in this study, dose-finding pharmacokinetic simulations were conducted to achieve similar AUC levels for healthy women and healthy males as modeled in the original study. Doses are based on pharmacokinetic simulations of a single 100 mg metoprolol dose using 1000 simulated time points throughout the course of a 24 h period. The results showed that a 100 mg dose of metoprolol in healthy young men produced the following PK parameters: AUC = 394 ng/mL·h, Cmax = 80.9 ng/mL, Tmax = 1.35 h, and T_1/2_ = 2.9 h. However, in women, the same 100 mg dose resulted in the following PK parameters: AUC = 967 ng/mL·h, Cmax = 134.5 ng/mL, Tmax = 1.44 h, and T_1/2_ = 4.3 h. A 50 mg dose in women results in AUC = 483 ng/mL·h, Cmax = 67.2 ng/mL, Tmax = 1.44 h, and T_1/2_ = 4.3 h. Thus, based on the dose-finding simulations, a 100 mg metoprolol dose in healthy young men will result in a similar systemic drug exposure, measured as AUC, to that of a 50 mg metoprolol dose in healthy young women. Results of the stochastic patient populations for men and women are shown in Figure 3.

### 3.3. Clinical Trial Simulations

**Figure 4:**
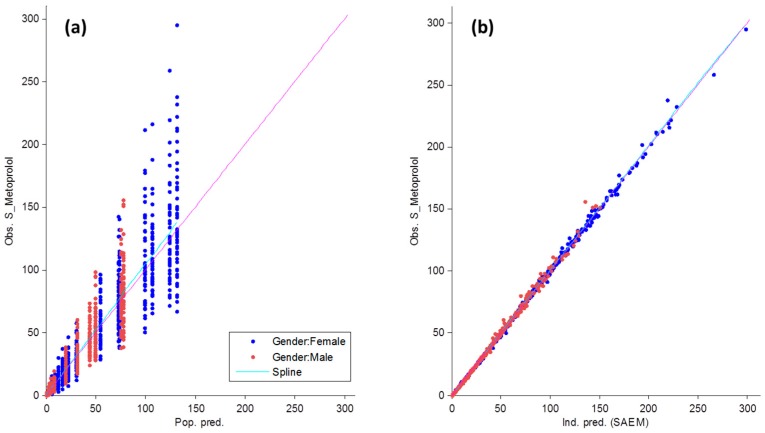
Goodness-of-fit plots for the observed versus predicted model diagnostics of the population (**a**) fit and the individual (**b**) fit of metoprolol plasma concentrations in healthy young men and women. The *x*-axis in (a) is the population predicted plasma concentrations while the *x*-axis on the right (b) illustrates the individual predicted concentrations based on the Stochastic Approximation Expectation–Maximization (SAEM) algorithm. The *y*-axes are the observed metoprolol concentrations.

**Figure 5:**
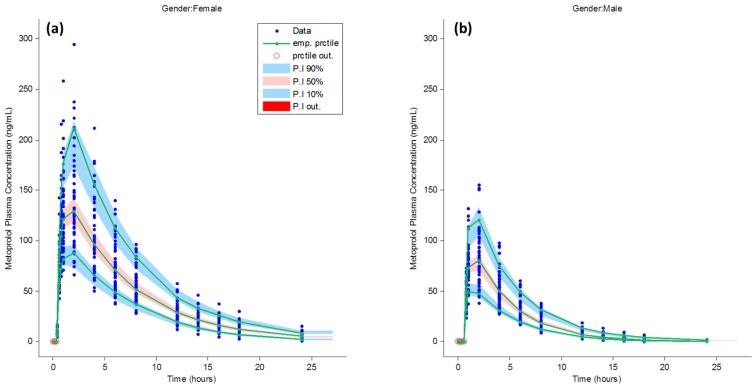
The prediction-corrected visual predictive check (PC-VPC) for a clinical trial simulation of metoprolol concentration–time plasma levels for healthy young women (**a**) and men (**b**). The shaded regions depict the 95% confidence intervals around the 10th, 50th, and 90th percentile range of plasma concentrations, while the solid line illustrates the average population pharmacokinetic metoprolol concentration. Emp. Prctile is the empirical percentile, prctile out is the percentile out, P.I. 90%, 50%, 10% and P.I. out are the 95% confidence intervals for the 10^th^, 50^th^, and 90^th^ percentiles while the P.I. out is the data predicted percentile out of the PC-VPC prediction interval(s).

A CTS of 100 patients that included gender-stratified concentration–time profiles that comprise 50 men and 50 women administered single 100 mg doses of *S*-metoprolol is shown in Figure 2. The Monte-Carlo simulations resulted in 1700 total plasma samples, with 850 metoprolol plasma levels for each male and female patient group. Results of the pharmacometrics analysis depicting the goodness-of-fit plots are shown in Figure 4. The final one-compartment model estimates that include the inter-individual variability, represented as the parameter variance (ω), are shown in Table 2. Model validation using the prediction-corrected visual predictive check (PC-VPC), which illustrates the gender-stratified covariate sub-population distributions, is shown in Figure 5.

## 4. Discussion

In evaluating the gender-stratified PK model parameters, men exhibit a quicker absorption rate and a longer time lag when compared to women. Furthermore, women exhibit a slower metoprolol clearance rate (women: CL = 101 L/h; men: CL = 253 L/h), as well as a smaller volume of distribution of metoprolol, when compared to men. I originally hypothesized a 30–40% dose reduction would be required in women; however, the results suggest the requirements for women would be a 50% dose reduction. Thus, model-based dosing simulations showed that a 100 mg dose in healthy young men would be equivalent to a 50 mg dose in healthy young women. In another study that evaluated metoprolol pharmacokinetics in geriatric participants, it was found that a 100 mg dose of metoprolol in healthy young men, who were CYP2D6 Extensive Metabolizers, resulted in similar total metoprolol drug exposures to a 50 mg dose in geriatric men and a 25 mg dose in geriatric women [1].

These results provide insight and guidance to physicians, pharmacists, and regulatory agencies for a potential modification of the metoprolol package insert to account for the effect of gender on dosing the cardio-selective β-blocker. Additionally, even though these results are not based on a patient-specific genotype status (e.g., CYP2D6 Poor Metabolizer) that would generally result in decreased clearance of a therapeutic agent and increased toxicity; these gender-based dosing recommendations may help support and align with the national Precision Medicine Initiative, by individualizing doses, by accounting for gender when dosing metoprolol. Personalized Medicine implementation strategies are successfully being initiated and employed throughout the United States and globally with the aims of decreasing drug toxicity and increasing efficacy to prescribed drugs by recommending dose adjustments on the basis of genotype [14]. However, accounting for both gender and genotype improve dosing recommendations to target therapeutic endpoints for our national endeavour to improve patient care by using principles of pharmacokinetics and pharmacodynamics used in clinical pharmacology.

When considering the differences in the AUC between men and women, it is important to note that body weight leading to direct influences to the hepatic tissue capacity of the metabolizing enzymes (i.e., CYP2D6), as well as sex-gender related hormones (e.g., estrogen and testosterone), influence the kinetics of all drugs. More specifically, in the original study, the average body weight ± standard deviation (SD) was 83.9 ± 10.7 kg in men and 62.0 ± 7.3 kg for the women included in the study [2]. Thus, the 21 kg difference in body weight may be discussed as the primary reason for the differences in the peak plasma levels of metoprolol. However, the point of body weight alone does not account for the clear anatomical size, physiological, and biochemical (e.g., testosterone and estrogen) differences between men and women. In men, the increased physical capacity of the left ventricle leading to a larger cardiac output (L/min), which in turn increases hepatic blood flow and metabolism rates in men, are a factor. Further, the same increase in cardiac output increases renal artery blood flow, and the clearance of xenobiotics is also an influential factor. Overall, despite variables that account for gender differences in the pharmacokinetics of metoprolol, the findings in this article show a 50% dose reduction results in equivalent metoprolol exposure to men and may help to explain and remedy the findings from studies reporting women who experience greater adverse effects related to cardiovascular medications than men [6,15,16]. With the hopes of precision medicine, dose adjustments based on gender, genotype, or both will become increasingly sought after by clinicians in medical practice and patients being administered these medications.

## 5. Conclusions

Overall, the findings of this analysis provide valuable information to clinicians: when prescribing a 100 mg dose of metoprolol for men, a 50 mg dose will be more appropriate for women. These results are based on pharmacokinetic modeling and simulations, and dose adjustments are aimed to avoid unnecessary doubling of the systemic exposure of metoprolol by accounting for gender.

## Figures and Tables

**Table 1:** One-compartment pharmacokinetic parameters for *R*- and *S*-metoprolol for young men and women.

	*S*-Metoprolol		*R*-Metoprolol	
	Female	Male	Female	Male
**V (L): Volume of distribution**	34.9	55.3	38.1	63.9
**CL (L/h): Clearance Rate**	101	253	120	316
**Ka (h^−1^): Absorption rate constant**	0.161	0.241	0.165	0.234
**Tlag (h): Absorption lag time**	0.38	0.67	0.39	0.59

**Table 2:** Gender-stratified population pharmacokinetic model estimates from the Clinical Trial Simulation (CTS).

Parameters	Men				Women			
	Value	SE	RSE (%)	CV (%)	Value	SE	RSE (%)	CV (%)
**Tlag (h)**	0.677	0.0021	0	0.20%	0.38	0.00013	0	0.20%
**Ka (1/h): Absorption Rate**	0.233	0.0058	2	42%	0.149	0.0037	2	42%
**V (L): Volume of Distribution**	49	1.5	3	43%	33.3	0.88	3	43%
**CL (L/h): Clearance Rate**	231	10	4	55%	92.9	4	4	55%
**Interindividual variability**								
**ωTlag, variance for Tlag**	0.0003	0.0021	809					
**ωKa, variance for Ka**	0.176	0.012	7					
**ωVd, variance for V**	0.182	0.013	7					
**ωCL, variance for CL**	0.305	0.022	7					
**Proportional error model**	0.0281	0.0007	2					

SE: standard error; RSE: relative standard error; CV: coefficient of variance.
